# Management strategies for implementing a multicenter cross-sectional study: lessons from the ADHERE Brazil study

**DOI:** 10.1590/1516-3180.2021.0492.R1.15092021

**Published:** 2022-04-02

**Authors:** Elisa Oliveira Marsicano-Souza, Fernando Antônio Basile Colugnati, Barbara Bruna Abreu de Castro, Maria do Socorro Van Keullen, Sabina De Geest, Helady Sanders-Pinheiro

**Affiliations:** I RN, PhD. Professor, Renal Transplantation Unit, University Hospital, Universidade Federal de Juiz de Fora (UFJF), Juiz de Fora (MG), Brazil; II PhD. Statistician and Professor, Renal Transplantation Unit, University Hospital, Universidade Federal de Juiz de Fora (UFJF), Juiz de Fora (MG), Brazil; III PhD. Biologist, Renal Transplantation Unit, University Hospital, Universidade Federal de Juiz de Fora (UFJF), Juiz de Fora (MG), Brazil; IV RN, MSc. Nurse, Renal Transplantation Unit, University Hospital, Universidade Federal de Juiz de Fora (UFJF), Juiz de Fora (MG), Brazil; V RN, PhD. Professor, Department of Public Health, Institute of Nursing Science, University of Basel, Basel, Switzerland; VI MD, PhD. Professor, Renal Transplantation Unit, University Hospital, Universidade Federal de Juiz de Fora (UFJF), Juiz de Fora (MG), Brazil

**Keywords:** Patient compliance, Multicenter study as topic, Kidney transplantation, Health information management, Data collection, Chronic patient healthcare, Epidemiological studies, Research operational issues, Research coordination, Limited research resources

## Abstract

**BACKGROUND:**

Epidemiological studies involving large samples usually face financial and operational challenges.

**OBJECTIVES:**

To describe the planning and execution of ADHERE Brazil, an epidemiological study on 1,105 kidney transplant patients, and report on how the study was structured, difficulties faced and solutions found.

**DESIGN AND SETTING:**

Cross-sectional multicenter study in 20 Brazilian kidney transplantation centers.

**METHODS:**

Actions developed in each phase of implementation were described, with emphasis on innovations used within the logistics of this study, aimed at estimating the prevalence of nonadherence to treatment.

**RESULTS:**

Coordination of activities was divided into four areas: general, regulatory, data collection and statistics. Weekly meetings were held for action planning. The general coordination team was in charge of project elaboration, choice of participating centers, definition of publication policy and monitoring other coordination teams. The regulatory team provided support to centers for submitting the project to ethics committees. The data collection team prepared a manual on the electronic collection system, scheduled web meetings and was available to respond to queries. It also monitored the data quality and reported any inadequacies found. Communication with the centers was through monthly reports via e-mail and distribution of exclusive material. The statistical team acted in all phases of the study, especially in creating the data analysis plan and data bank, generation of randomization lists and data extraction.

**CONCLUSIONS:**

Through these logistics, we collected high-quality data and built a local research infrastructure for further studies. We present supporting alternatives for conducting similar studies.

**CLINICAL TRIAL ANNOTATION:**

http://clinicaltrials.gov/ on October 10, 2013; NCT02066935.

## INTRODUCTION

Nonadherence to treatment of chronic diseases is one of the main determinants of long-term complications.^1^ Kidney transplantation (KT) is one of the therapies used for the most advanced phase of chronic kidney disease. It results in better survival, better quality of life and lower costs.^2^ Nonadherence to immunosuppressive drugs that are necessary to avoid graft rejection is associated with an increased risk of acute rejection episodes, graft dysfunction, lower graft survival and higher healthcare costs.^2^

Over recent years, Brazil has reached the second highest position in the world, in terms of the absolute number of KTs performed in the world’s largest public transplantation system.^3^ However, this country has a large geographical size and great cultural, social and economic diversity, which translates into large variation in transplantation activity across the country, when normalized according to population size.^4^ Evaluation of and addressing nonadherence after KT is crucial to achieve the best results. However, Brazilian studies are scarce, limited to small samples, and are therefore not representative of the general population.^5,6,7,8,9,10,11,12^

Studies on adherence to immunosuppressives after KT by our group began in 2009. In the initial project, we performed a cross-cultural adaptation and validation study of a self-report instrument for diagnosing nonadherence to immunosuppressives.^8^ Then, we estimated the prevalence of and identified factors associated with nonadherence to immunosuppressives after KT in a single center in the city of Juiz de Fora, Minas Gerais.^10^ Discussion on the need for a multicenter study that could better portray Brazilian epidemiology arose when these results were presented in national conferences in 2011. Over a period of two years, these discussions continued and led to planning of a project among Brazilian national researchers in collaboration with a world-reference research center that had already started a similar study on heart transplantation (Building Research Initiative Group: Chronic Illness Management and Adherence in Transplantation - BRIGHT study).^13^ Consequently, the ADHERE Brazil study was designed as a multicenter cross-sectional study with the following objectives: to estimate the prevalence of nonadherence to immunosuppressives and other aspects of nonpharmacological treatment; to evaluate multilevel factors associated with nonadherence to treatment; and to benchmark the participating centers, bring the subject up for discussion and disseminate data to support future actions towards reducing these behaviors.^14^

However, conducting such a large study, comprising twenty kidney transplantation centers and an ideal sample size of 1,139 patients, presented several challenges. This was an epidemiological study, and therefore without interventions, but which needed adequate infrastructure, in order to follow regulatory policies, and needed funding similar to that required in clinical research. For example, the study required a study coordination room, a meeting room and a specific area for archives and staff training.^15,16^

A multicenter study can be divided into four phases: 1) Planning; 2) Project development; 3) Study implementation; and 4) Dissemination. Some detailed reports on each of these phases exist, but the actions developed and the project management may vary according to the project and local characteristics.^17^ Indeed, in our project we faced some difficulties, but feasible solutions were found that translated into a path towards positive results. Considering that these challenges may be experienced in similar research proposals, a report presenting a detailed list of actions may help other researchers succeed.

## OBJECTIVE

Thus, our objective was to present the stages followed by the researchers responsible for conception of the ADHERE Brazil study; show how the development of the study was planned and implemented; and describe the difficulties found and solutions applied. Ultimately, reliable results could be obtained regarding the diagnosis of nonadherence to treatment and associated factors. We therefore present a description of the actions taken, especially in the project implementation phase, with emphasis on the innovative and adaptive processes employed.

## METHODS

This was a cross-sectional and descriptive study on the procedures undertaken for execution and management of the ADHERE Brazil multicenter study (http://clinicaltrials.gov/ on October 10, 2013; NCT02066935).

Use of project management principles through coordination teams, for the various phases of a study, is fundamental for enabling of a multicenter study to be properly conducted.^17,18^ Many of these studies are conducted by clinical research centers with professional support from companies that are in charge of managing all phases of the project.^19^ In the case of the ADHERE Brazil study, because of its epidemiological nature and its objective of being representative of all types of KT services, it was not envisaged that the project would have the infrastructure of the multicenter studies mentioned above. Accordingly, a specific and local proposal was developed.

Here, we describe the organizational structure proposed at different phases of our study for actions to be elaborated, especially in the project implementation phase, so that it would be possible to reproduce in similar studies. This report was authored by the researchers who participated in the study.

## RESULTS

The initial idea for the project arose in 2011. It was then designed over a two-year period. A group of co-investigators, whom we called the ADHERE Brazil study consortium, was then consolidated. The project was registered at the Research Department of the Federal University of Juiz de Fora, the institution to which the principal investigator was attached; and on the Clinical Trials website (https://clinicaltrials.gov/) under the number NCT02066935 and on the Open Science Framework scientific dissemination platform (https://osf.io/dpr2j/).

**Figure 1.**
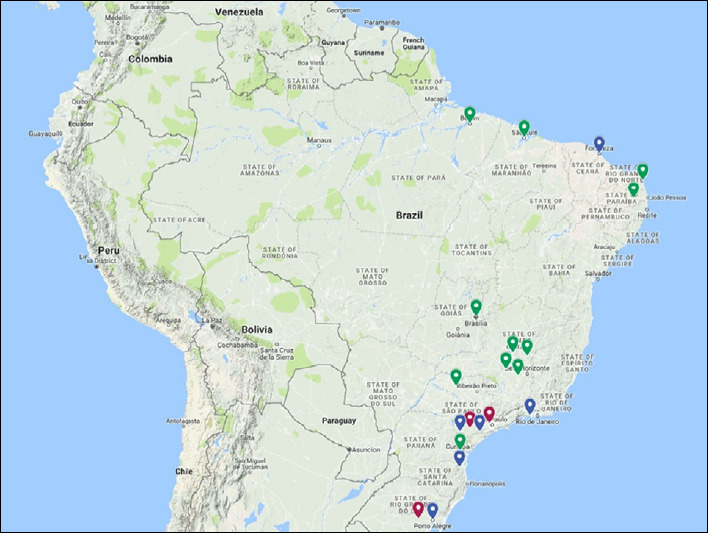
Geographical locations of participating centers in the ADHERE Brazil study around the country. Colors indicate the transplantation activity level: red, high activity (> 150 kidney transplantations/year); blue, moderate activity (50 to 150 kidney transplantations/year); and green, low activity (< 50 kidney transplantations/year).

Recruitment of potential study participant centers began in 2013. The initial invitations to 20 centers were made in person at national and regional conferences. After communicating by e-mail and signing statements of confidentiality and feasibility, the participation of these centers was formalized through a contract between them and the principal investigator’s center (coordinating center). Invitations were sent out in May 2013, and the final composition of the participating centers was completed in May 2016. A total of 22 centers were invited because two of the initially invited centers subsequently refused to participate. The new invitations were made using the same criteria and characteristics as for the original centers. Among the 20 centers that ultimately participated in the ADHERE Brazil study, 38.2% showed low activity (< 50 transplantations/year), 36% were moderately active (50-150 transplantations/year) and 25.8% were highly active (> 150 transplantations/year). This distribution was similar to what had been reported by the Brazilian Transplant Register^5^ (Figure 1).

Scientific participation and authorship were defined before the study started and were described and agreed upon in a publication policy statement. The regulatory and fundraising phases began in 2014. The first sets of data were collected in December 2015 and finalized in April 2017. Dissemination of results began in 2015 and is ongoing. By April 2021, 17 presentations had been made in national/regional conferences and five in international events, and two articles had already been published.^14,20^

As mentioned above, we established an early research collaboration with the Leuven Basel Research Group (LBARG). They already had broad experience in international dissemination of knowledge and multicenter studies. The group supported all the phases of our study, through a strengthened partnership and promotion of a better qualified process.

To make the project feasible, coordination of actions in four areas was proposed: general, regulatory, data collection and statistics.

### General coordination

General coordination was performed by the principal investigator of the study, who was responsible for project elaboration, study consortium formation and sending invitations to the participating transplantation centers. She also participated in all the other phases: regulation, data collection and analysis. In addition, she prepared other reference documents for the study, such as the statements for center participation feasibility and confidentiality, as well as the publication policy of the study.

In the center recruitment phase, after the informal invitation had been accepted, the centers received a confidentiality agreement, which addressed the importance of confidentiality of the project content and of the data collected during the study. After signing, the center received the feasibility document, which contained information that would ensure that the participating center had the minimum infrastructure to be able to conduct the study. The center also received the publication policy statement on issues relating to publications generated using ADHERE Brazil study data, authorship and subprojects. Centers were only included in the study after the leading local investigator of each center signed these documents. After the regulatory phase, participation was formalized through a research contract signed by the parties involved.

Soon after regulatory approval had been obtained, applications for financial support were sent out. Support was sought from the national governmental funding agency (Conselho Nacional de Desenvolvimento Científico e Tecnológico, CNPq) and from the local state agency (Fundação de Amparo à Pesquisa do Estado de Minas Gerais, FAPEMIG); and also from private companies interested in research relating to transplantation. Unfortunately, the project was not selected for governmental support, but two pharmaceutical companies decided to join the proposal. The operational support provided by a clinical research structure and the virtual and centralized data collection system, together with interest in the potential results, seemed to contribute to the companies’ decision. However, other than financial support, the companies had no role in any of the stages of the project design, data collection, analysis or writing of the manuscripts. The resources enabled personnel payments (regulatory and data collection activities), participation in events, translation/editing and printing services.

**Figure 2.**
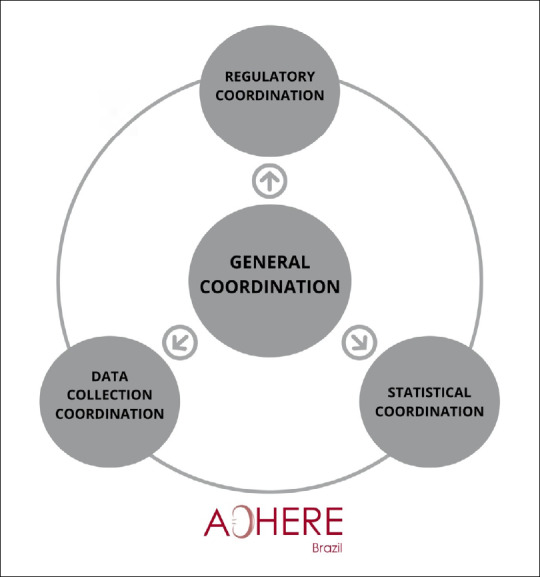
Organizational structure, showing the four coordination teams and flux process among them.

Weekly meetings were held among the general coordination team and the other coordinators to schedule the following phases of the study: forwarding the participating center projects to the local ethics committees; organizing and training for data collection; checking the ongoing data collection; and performing data analysis. This coordination team was responsible for following up on all the activities necessary for development of the project, with organization and control of the information (Figure 2 and Table 1).

**Table 1.** Description of the participation of each coordination team at different phases of the ADHERE Brazil study

Phases Coordination team	Phase 1 Regulation	Phase 2 Data collection training	Phase 3 Data collection	Phase 3 Data analysis
General	X	X	X	X
Regulatory	X			
Data collection		X	X	
Statistical		X	X	X

The general coordination effectively developed the following strategic activities for project progression: 1) Weekly meetings mentioned above; 2) Definition of well-established functions among the other coordination teams; 3) Creation of an e-mail address shared by the coordinators; 4) Communication with the centers via Skype or other communication sources such as telephone or e-mail, to answer questions; 5) Monthly newsletters to the centers, which totaled 28 between 2015 and 2020, reporting on study progression; 6) Meetings with members of the participating centers at national conferences to disseminate information about the study proposals (14^th^Brazilian Congress on Transplantation 2015 and 17^th^Brazilian Congress of Nephrology 2016), along with distribution of material containing the visual identity of the study (buttons, notebooks and pens) (Table 1); 7) Provision of an appropriate physical area for meetings, a telephone line and a computer for coordinators to work on.

### Regulatory coordination

Initially, the coordinating center submitted the project to the research ethics committee (REC) of the institution at which that center was located. After approval, which was granted in May 2014, the centers recruited were asked to submit the project to their local RECs. Participation of each center depended on receiving approval from the local REC. Patients were only included after they had signed an informed consent statement (ICS).

The first step for the coordinator of the regulatory team was to send all the guidelines for REC submission to the other 19 participating centers and a model of the formal documents to be included in the national REC website (Brazil Platform): research project, ICS model, budget model and schedule model. This guidance greatly facilitated REC submission by the participating centers. However, there were some difficulties, which can be gauged from the time that elapsed between obtaining the first REC approval (September 2014) and obtaining the last one (February 2017), which amounted to 30 months.

The main difficulty faced was the lack of familiarity with the Brazil Platform among the participating researchers, with regard to registering researchers, preparing documents to be included and, subsequently, formulating answers to any questions raised by the RECs. For these points, availability and participation of the regulatory team was essential for solving any issues. Another difficulty was the refusal of two RECs to evaluate the project because it was a multicenter study. These cases were then referred to and evaluated by the coordinating center’s REC. This process delayed patient inclusion in these centers. Lastly, after initial approval of the project, two amendments for replacement/inclusion of two new centers had to be submitted to all RECs. The archiving of documents submitted to each local REC and final approvals from them was done by the regulatory team.

It was also the responsibility of this coordination group, after the end of data collection, to forward the final report to the coordinating center REC and to send a model to the participating centers, for them to perform the same procedure in local RECs.

This team was also responsible for registering the project on the Clinical Trials website, which ensured transparency with regard to execution and publication of results and avoidance of bias, with ethical support for the participants, since the results generated would promote scientific knowledge.^21^

### Data collection coordination

The two-member data collection coordination team oversaw organizing the training of personnel involved in data collection at the participating centers and monitoring and checking data inclusion during collection. The objective of this coordination team was to make available staff who had been trained to operate the data collection system, on the Research Electronic Data Capture (RedCap) platform, and who would be easily accessible to people at the centers, by telephone, e-mail or other communication platforms.

The interviews and questionnaires used in the ADHERE Brazil study were prepared based on a theoretical framework^1,14^ and on previous studies.^13^ The RedCap system was used for data collection. This is a safe internet-based software platform that was created at Vanderbilt University, in Nashville, United States, for the purposes of data capture and storage, which could be fed remotely by trained individuals (http://www.project-redcap.org/) at the coordinating and participating centers. The ADHERE Brazil project was created on this platform; thereafter, the data collection questionnaires were included. Each center had remote access to the project to include data and access only their respective data, thereby ensuring anonymity.

All the research coordinators of the centers participating in the ADHERE Brazil study were trained to use the RedCap system through a manual that was prepared specifically for this purpose, and through Skype meetings. In addition to training, each center was asked to include a “test patient” in the system before data collection started.

Another data collection coordination function was to send randomization lists that had been generated by the statistics coordination team, to the participating centers so that patients could be selected to participate in the study.

Lastly, to ensure data quality, the data collection team checked the data weekly, as the patients were inserted into the RedCap system by the participating centers, to detect missing data or possible typing errors. Following this evaluation, reports were generated every week and sent to the centers, requesting them to check and correct inconsistencies if necessary. A one-week deadline was given for checking the data. Thereafter, the data were checked again.

One of the biggest challenges of the data collection coordination team was to adjust for missing/inconsistent data from each center. Data collection was only considered finalized after inconsistencies had been checked and corrected. To solve such problems after a report had been sent more than once, telephone contact was made to detect possible process difficulties. Using this strategy, data collection began in December 2015 and was completed in April 2017 (a total of 17 months), with inclusion of 1,105 patients, which corresponded to 97% of the ideal sample size.

### Statistical coordination

The statistical coordination team participated in all study phases, from conception to analysis and dissemination of data.

More specifically, it was responsible for the sampling strategy, and for defining the sample size and the criteria for patient distribution in each center. It created the database in the RedCap system and sent access passwords to the 20 participating centers. RedCap access levels differ, depending on the function: the centers only had access to their own information, while the data collection and statistics coordination teams and the principal investigator had broader access. Additionally, the statistical coordination team was responsible for generating randomization lists using computer software to select patients for the study. These lists included information provided by the centers, such as the number of patients scheduled per day in the post-transplantation clinic and the number of patients to be included in each period.

Another function of this coordination team was to organize data extraction after data collection through the RedCap system had been completed, thus generating the initial ADHERE Brazil study database. The entire analysis plan was defined by this coordination team in agreement with the researchers’ consortium. It was also responsible for defining and guiding the tests performed and for performing more complex analyses.

## DISCUSSION

The ADHERE Brazil study involved data collection from 20 Brazilian KT centers. Active participation by the general, regulatory, data collection, and statistical coordination teams was essential for enabling effective collection and reliable analysis of the information generated. Consequently, these integrated actions enabled inclusion of 97% of the ideal sample size, which was accomplished with high-quality data collection and integration among the coordination teams. The strength of our study that we can highlight is that we were able to accomplish a high-complexity study using low-cost available solutions, thus generating unpublished data with great potential to contribute towards healthcare practice.

The general coordination team conducted activities in conjunction with the other coordination teams in a harmonious fashion. Based on the basic principles of project management in research,^21^ weekly updates with discussion of the problems that arose, together with short and medium-term planning of solutions, aligned project progression and established the actions and functions to be fulfilled.^21^ This proposal was essential for solving problems in our study, as previously described in health and educational research settings.^21–24^ Management skills among health researchers are not fully available because such abilities are not systematically developed during undergraduate courses. Additionally, investigators are frequently involved in other activities such as teaching or healthcare assistance outside of research, and thus have limited time to dispend on further educational and training efforts.^25^

In line with international standards,^17^ all research in Brazil involving human beings has to be evaluated by a REC to ensure respect for and protection of research subjects.^21^ The role of RECs is to ensure that the principles of research ethics are respected in the study and that the rights of those involved are preserved.^21^ Although there is clear legislation on regulatory procedures for this type of research, the effectiveness and operational particularities of RECs still vary across Brazil.^26,27^ For multicenter studies, which involve multiple REC evaluations, some avoidable inefficiencies have been reported, mainly due to discrepancies in the opinions of these multiple committees.^27^ This can be demonstrated through the long period of time that was spent on this phase, which was the longest. The regulatory coordination team was created to deal with these issues, and to promote and facilitate regulatory procedures in each center, since the study included some institutions with fully functioning clinical research units and others that had never participated in research. We proposed to have someone with expertise in regulatory processes always available to assist at all levels, from preparation of documents to submission of the project and its amendments to the Brazil Platform website. A similar strategy has been reported by others in multicenter studies.^17,23,28^

The data collection phase is essential. Good planning at this stage can prevent possible distortions and interviewer influence on the interviewee, which thus enables methodological and scientific rigor.^17,19,22,23^ It is important to emphasize that the quality of the data collected in surveys depends on the adequacy and quality of questionnaires, in order to guarantee validity and reproducibility through clear and simple questions.^16,17^ Furthermore, to increase survey quality, it is essential to provide standardized training for data collectors.^17,23^ In the ADHERE Brazil study, all professionals who collected data received training for this purpose. Another key feature was simultaneous data collection and storage through a computer platform connected to the internet, which minimized errors, since it allowed for immediate checking for missing or erroneously filled data. These errors could be reported back to the person responsible for data collection, so that prompt corrections could be requested. Systematic and consistent checking of quality during the ongoing data inclusion is a powerful measure for preventing errors and missing data.^17^ In our study, each local center made adaptations and took appropriate steps to ensure that data were collected in a way that guaranteed satisfactory results.^17,23^

It is worth mentioning that it is the responsibility of statisticians to plan studies, interpret the data obtained through field research and present the results in a way that facilitates decision-making by researchers.^15,16,28^ High-quality research is associated with formal early statistical planning.^29^ Thus, it is essential to have statisticians’ participation at all stages of a research project so that they can significantly contribute not only to data analysis, but also to the choice of method and analysis software, and to presentation and interpretation of results.^29^ The availability and effective participation of the statistical coordination team at all levels of our study led to attainment of this quality threshold.

The limitation of this study was the scarcity of articles describing experiences relating to strategies for conducting multicenter epidemiological studies. Especially within the Brazilian scenario, we found reports only about obstacles to the ethics regulatory process.^26,27^ This made it difficult to discuss the key points of the proposal from a local perspective. Therefore, we discussed the findings based on the available published reports. We think this aspect of our proposal highlights its originality and relevance.

Since the conceptualization of the ADHERE Brazil study, we have been making efforts to disseminate the proposal and the results. Our proposal was registered on the Clinical Trials and the Open Science Framework websites, and we have presented summaries of our results at international, national and regional meetings. In confirmation of the potential for applicability and reproducibility of our proposal, we have already identified two Brazilian multicenter studies that mirror our methodology: the DGF Brazil Study Group^30^ and SARS-COV-2 Infection in Kidney Transplant Recipients: a Brazilian Multicenter Study (http://clinicaltrials.gov/: NCT04494776).

## CONCLUSION

The experience acquired in conducting this study led us to conclude that through the project management actions described, it was possible to collect reliable data on adherence to immunosuppressive treatment after KT and to ensure that the ethical principles and safety measures involved in the research and data collection were adopted, since all work processes proposed for conducting the study were strictly followed. In addition, this article presents alternatives for conducting studies of similar nature to the ADHERE Brazil study.
